# Debating Frontline Therapy in Chronic Myeloid Leukemia

**DOI:** 10.3389/fonc.2021.708823

**Published:** 2021-09-08

**Authors:** Xia Bi, Sabarina Ramanathan, Gina Keiffer

**Affiliations:** Department of Medical Oncology, Thomas Jefferson University, Philadelphia, PA, United States

**Keywords:** CML - chronic myelogenous leukemia, imatinib, second generation tyrosine kinase inhibitors, TKI - tyrosine kinase inhibitor, dasatinib, nilotinib, bosutinib, ponatinib

## Introduction

The development of tyrosine kinase inhibitors (TKIs) in the early 2000s revolutionized the therapeutic landscape for chronic phase (CP) chronic myeloid leukemia (CML). Interferon alpha was previously the standard treatment for patients with CP CML prior to the development of TKIs; however, survival was dismal, with a median of 5-6 years (1). With the advent of TKI therapy, the estimated 10-year overall survival increased from less than 20% to more than 80% (2). When optimal therapy is instituted with appropriate monitoring, patients with CP CML now live close to normal life spans (3, 4).

Imatinib, the first of these revolutionary medications, was approved by the FDA for the treatment of CML after failure of interferon-alpha therapy in 2001 and for newly diagnosed CP CML patients in 2003. Shortly thereafter, following preclinical and clinical data demonstrating increased potency against the BCR-ABL target, frontline approvals for the second-generation TKIs (dasatinib, nilotinib, and bosutinib) soon followed based on the results of their respective clinical trials (DASISION, ENEST and BFORE) (5–7). Each of these trials compared the efficacy of imatinib 400 mg daily with the corresponding second-generation TKI. Notably, no significant survival difference has been demonstrated between imatinib and any of the second-generation inhibitors. Accordingly, the European Society for Medical Oncology (ESMO) and the National Comprehensive Cancer Network (NCCN) practice guidelines recommend choosing a therapeutic agent based on risk scoring, age, and comorbidities (8, 9). Thus, the optimal frontline treatment of CP CML has become the subject of debate.

A recently published meta-analysis sought to answer this question by comparing the safety and efficacy of imatinib versus dasatinib, nilotinib, bosutinib and ponatinib for initial treatment of CP CML (10). Second- and third-generation TKIs demonstrated superior clinical outcomes but also increased toxicity. The authors concluded that the choice of frontline therapy should depend on patients’ age and comorbidities. They suggested that patients without comorbidities should receive second-generation TKIs as initial therapy and that imatinib should be the preferred initial therapy for older patients or those with comorbidities. Notably, third-generation TKI (ponatinib) has not been approved nor is recommended as the frontline treatment of CP AML. Herein, we will review this recently published work and present the arguments for and against the use of second-generation TKIs as initial therapy for CP CML.

## The Data

In this meta-analysis, the authors systematically reviewed randomized controlled trials (RCTs) that compared the efficacy and safety of imatinib *vs* second-generation (dasatinib, nilotinib, bosutinib) or third-generation (ponatinib) TKIs in adults with newly diagnosed Philadelphia chromosome-positive (Ph+) CP CML. Nineteen relevant studies and 15 relevant abstracts published between 1990 and 2019, corresponding to 7 RCTs involving 3262 participants, were included. The primary outcomes were overall survival (OS) and progression-free survival (PFS). Secondary outcomes included various efficacy and safety measures.

There was no statistically significant difference in the primary outcomes, although only 2 of the 7 RCTs reported OS and PFS up to 60 months and only 1 reported OS up to 72 months. In terms of secondary outcomes, all of the pooled efficacy outcomes except for drug discontinuation showed a clear advantage of second- and third-generation TKIs over imatinib. **Table 1** summarizes the relative risks (RRs) of later-generation TKIs in comparison with imatinib in terms of efficacy and toxicity as reported in the ENEST, DASISION, and BFORE studies and in the pooled analysis. The RR of major molecular response (MMR) after 3 months was statistically higher than all other efficacy outcomes in patients treated with later-generation TKIs (RR = 4.50; 95% confidence interval [CI], 2.23-9.09). In terms of adverse events, there were more cases of thrombocytopenia, cardiovascular events, pancreatic and hepatic effects in patients treated with later-generation TKIs. Specifically, hepatic effects had the highest RR in the bosutinib group.

**Table 1:** Relative risk of later-generation TKIs in comparison with imatinib by efficacy and safety endpoints.

		ENEST*	DASISION	BFORE	Pooled*
		RR (95% CI)	RR (95% CI)	RR (95% CI)	RR (95% CI)
**Efficacy endpoints**					
EMR at 3 months	**1.36 (1.24-1.49)**		**1.31 (1.17-1.46)**	**1.31 (1.15-1.50)**	**1.34 (1.27-1.41)**
MMR at 3 months	**8.36 (2.55-27.38)**		NR	2.45 (0.78-7.70)	**4.5 (2.23-9.09)**
MMR at 12 months	**1.90 (1.55-2.33)**		**1.49 (1.17-1.92)**	**1.28 (1.03-1.58)**	**1.52 (1.32-1.75)**
CCyR at 12 months	**1.22 (1.14-1.32)**		**1.17 (1.06-1.28)**	NR	**1.13 (1.04-1.22)**
CCyR by 12 months	**1.23 (1.11-1.37)**		**1.16 (1.06-1.27)**	**1.16 (1.04-1.30)**	**1.15 (1.09-1.22)**
MR4 at any time	**2.44 (1.62-3.67)**		**1.39 (1.08-1.78)**	**1.72 (1.13-2.62)**	**1.67 (1.32-2.11)**
MR4.5 at any time	**3.38 (1.76-6.48)**		1.39 (0.99-1.94)	**2.45 (1.10-5.45)**	**2.65 (1.44-4.88)**
AP/BP during study	**0.17 (0.04-0.74)**		0.62 (0.26-1.47)	0.66 (0.19-2.31)	**0.43 (0.25-0.73)**
**Safety endpoints**					
Anemia	0.61 (0.30-1.27)		1.48 (0.90-2.44)	0.74 (0.32-1.73)	1.17 (0.80-1.72)
Neutropenia	**0.56 (0.38-0.82)**		1.21 (0.91-1.61)	**0.56 (0.32-0.97)**	0.69 (0.46-1.02)
Thrombocytopenia	1.16 (0.70-1.94)		**1.58 (1.08-2.32)**	**2.44 (1.37-4.34)**	**1.55 (1.17-2.05)**
Cardiovascular events	2.61 (0.94-7.22)		2.25 (0.70-7.21)	1.98 (0.50-7.83)	**2.26 (1.32-3.87)**
Cutaneous effects	0.40 (0.08-2.05)		0.09 (0.01-1.64)	0.33 (0.03-3.15)	0.73 (0.21-2.47)
Gastrointestinal effects	0.59 (0.28-1.27)		0.14 (0.01-2.75)	**3.19 (1.54-6.60)**	1.80 (0.67-4.84)
Fluid retention	9.03 (0.49-166.97)		15.00 (0.86-261.28)	2.97 (0.12-72.49)	**3.21 (1.09-9.48)**
Infectious events	3.01 (0.12-73.59)		5.00 (0.59-42.50)	0.68 (0.30-1.57)	1.11 (0.54-2.28)
Pancreatic effects	**2.09 (1.07-4.08)**		NE	1.84 (0.98-3.44)	**2.24 (1.29-3.87)**
Hepatic effects	1.72 (0.69-4.31)		0.25 (0.03-2.22)	**5.84 (3.16-10.82)**	**3.01 (1.21-7.51)**
Musculoskeletal disorders	0.25 (0.03-2.23)		NE	0.82 (0.25-2.67)	0.76 (0.36-1.62)
QT prolongation	0.50 (0.09-2.72)		0.70 (0.27-1.81)	0.99 (0.06-15.73)	0.82 (0.39-1.73)

EMR, early molecular response (Bcr-Abl IS = 10%); MMR, major molecular response (Bcr-Abl IS = 0.1%); CCyR, complete cytogenetic response; MR4, Bcr-Abl IS = 0.01%; MR4.5, Bcr-Abl IS = 0.0032%; AP/BP, accelerated phase/blast phase transformation.

NR, not reported; NE, not estimable.

Bold = statistically significant.

*Data for nilotinib 300mg twice daily.

## Point: The Case for Using Second Generation TKIs as Initial Therapy for CP CML

The meta-analysis demonstrated a statistically significant improvement of 3-month MMR and other efficacy outcomes in patients treated with second- and third-generation TKIs. Attainment of an early molecular response (EMR; BCR-ABL1 IS ≤10% at 3 months) has been shown to be an important treatment milestone in patients with newly diagnosed CP CML (11–13) and is the first benchmark for evaluating responses to TKI therapy (9, 14). Failure to achieve an EMR suggests treatment failure, and consideration should be given to alternate therapy (9, 15). Previous studies showed superior PFS and OS in patients who were able to achieve this early molecular milestone, and that EMR failure was associated with lower rates of molecular response and increased risk of disease progression (16–19). Therefore, achievement of an EMR may predict long-term clinical outcomes and allow early intervention for patients who are less likely to respond to treatment.

Another important consideration in selecting an initial therapy is the ability to safely stop therapy and maintain a treatment-free remission (TFR). TFR is defined as maintaining a BCR-ABL1 IS ≤0.1% (MMR) off TKI therapy. In order to safely discontinue TKI therapy, patients are recommended to achieve and maintain a deep molecular response (DMR; BCR-ABL1 IS ≤0.01%) for ≥2 years (9). TFR is most successful in patients with at least 4 years of TKI therapy who achieve and maintain DMR for at least 2 years prior to treatment cessation (14, 20, 21). Successful TFR limits treatment-associated AEs, decreases cost and allows for fertility. Thus, the ability to maintain TFR is especially important for young patients and those who desire fertility.

In this meta-analysis, more patients treated with later-generation TKIs were able to achieve a MR4 and MR4.5 (RR = 1.64, RR = 2.63, respectively), which is associated with higher survival probabilities and greater chance of TFR (22). The ENEST study, with more than 10 years of follow up, demonstrated higher cumulative incidence of achieving MR4.5 for nilotinib-treated patients (61%) than imatinib-treated patients (39.2%) (23). Patients treated with nilotinib also had higher rates of TFR eligibility (48.6% *vs* 29.7%) (23), which supports the use of second-generation TKIs in patients aiming for TFR.

Treatment with later-generation TKIs is also associated with decreased progression to AP or BP (RR = 0.44, 95% CI 0.26-0.74), thus resulting in fewer patients needing intensive chemotherapy and stem cell transplantation and preventing the significant morbidity, mortality and cost associated with these therapies. This benefit was seen especially in patients with high risk Sokal scores, where only 7 (9%) patients treated with nilotinib 300mg twice daily experienced progression to AP and BP compared to 11 (14%) patients treated with imatinib in the ENEST study (24).

## Counterpoint: The Case Against Using Second Generation TKIs as Initial Therapy for CP CML

Imatinib was the first TKI to be approved by the FDA for patients with CML in all phases based on the results of the landmark IRIS study which compared the efficacy of imatinib with the standard of care interferon and cytarabine. After a median follow-up of 19 months, imatinib demonstrated significant improvement in rates of CCyR (74% versus 9%, P< 0.001) and freedom from progression to AP or BP at 12 months (99% versus 93%, P< 0.001) (25). Further follow-up at 10 years demonstrated that 93% of imatinib-treated patients achieved MMR, 63% achieved MR4.5 and, astoundingly, overall survival was 83.3%, establishing the long-term durability of imatinib (2).

Despite improvement the improvement in time to response and depth of response seen with second generation TKIs, they have never shown an overall survival or progression free survival benefit beyond imatinib. The 5‐year OS rates for nilotinib *vs* imatinib was 94% *vs* 92%, and dasatinib *vs* imatinib was 91% *vs* 90% (**Table 1**) (19, 24, 26). Outcomes stratified by disease risk score (Sokal, Euro/Hasford) similarly demonstrated no significant survival difference between nilotinib and imatinib (6). While the higher dose of nilotinib (400mg twice daily) showed superior OS and PFS, this dosage was associated with unacceptable levels of cardiovascular toxicity and is only employed in AP CML. Similarly, after 5 years of follow up of the BFORE trial, there was no differences in EFS in treatment arms and OS rates were comparable at 94.5% for bosutinib *vs* 94.6% for imatinib (27).

In addition to the lack of improvement in overall survival, second-generation TKIs have been shown to have increased toxicity compared to imatinib. All TKIs cause cytopenias and gastrointestinal (GI) side effects, but in clinical experience, most AEs for imatinib are mild and manageable while the newer agents portend greater early and later serious AEs and grade ≥3 AEs that lead to treatment discontinuation. The risk of vaso-occlusive events (VOEs) in particular is increased with second-generation TKIs. A meta-analysis pooling ten trials consisting of >3000 patients demonstrated that dasatinib, nilotinib, and ponatinib usage increased risk of vascular occlusive events. Events were observed in 5.8% of patients (93 of 1582) treated with second-generation TKIs *vs* 1.0% of patients (13 of 1253) on imatinib. The study reported significantly higher VOEs with nilotinib (odds ratio [OR], 3.45) and dasatinib (OR, 3.86) in comparison to imatinib (28).

Finally, for any potentially life-long therapy, the overall cost of treatment, including not only the cost of the treatment itself but of the additional cost of managing adverse effects and monitoring of therapy, must be considered in selecting an upfront treatment strategy. Cost-effectiveness analyses in the US and Japan simulating the clinical course of 10 years of CML treatment, mapped cost estimate probabilities starting with imatinib, dasatinib, nilotinib, or any TKI according to the physician’s choice. This model demonstrated a value advantage for imatinib-first sequential treatment strategies over the initial use of second-generation agents even after factoring in drug discontinuation at the 2-year DMR target (29). While the cost of imatinib has varied over the years, data suggests that generic imatinib could be purchased at a daily cost as low as $20 in 2018. Interestingly, despite the lack of survival data, the use of second-generation TKIs tripled from 19% to 56% from the years 2010 to 2019. In parallel, the daily cost of newer agents increased from $243 per day in 2010 to $354 in 2018 validating that individual and overall health care costs are higher with newer agents potentially causing higher out-of-pocket expenses, increased financial burden and treatment delays (30).

While maintaining deep and durable molecular responses remains the therapeutic goal for patients with CML, the ultimate goal of therapy is to help patients live better and longer. To this end, imatinib has demonstrated prolonged survival over sufficient observation periods as well as improved tolerability compared to second generation TKIs. Additionally, imatinib is generic and is more widely available than second generation TKIs and, despite few patients attaining TFR, imatinib has shown improved cost effectiveness beyond second generation TKIs. It is worth noting that second generation TKIs remain a very effective second-line therapy for patients who progress on or are intolerant of imatinib, allowing almost half of those patients to achieve a complete cytogenetic response (31).

## Conclusions

A first-generation TKI, imatinib and three second-generation TKIs, dasatinib, nilotinib, and bosutinib, have all been approved for the initial treatment of CP CML, prompting the question of which TKI is the best one to use as initial therapy. Although no additional OS or PFS benefit has been seen with the use of second-generation TKIs compared to imatinib, they have demonstrated superiority in several surrogate endpoints of clinical efficacy, including higher rates and depth of response and decreased progression into AP and BP. In the absence of an overall survival or progression free survival benefit, the decision for which TKI to use as initial therapy should take in to account individual patients’ medical comorbidities and the importance of attaining TFR, particularly for patients who desire fertility.

We propose using second-generation TKIs as frontline therapy in young patients, those who desire fertility and those with intermediate or high risk disease. Imatinib is more appropriate for low- to intermediate-risk disease, older patients, or those with other medical comorbidities as it is generally better-tolerated and has a good safety profile with the longest follow up. Affordability is key especially when treatment may continue throughout life. Imatinib still remains the most frequently used TKI around the world for various reasons, particularly because of lower cost, greater access, and familiarity. Regardless of which TKI is used as initial therapy, appropriate patient adherence, close monitoring of disease response, swift change in treatment for those who fail to meet treatment milestones and close monitoring for adverse effects is necessary to ensure treatment success.

## Author Contributions

XB and SR conducted research and drafted and revised this article. GK provided critical revision and final editing support. All authors contributed to the article and approved the submitted version.

## Conflict of Interest

The authors declare that the research was conducted in the absence of any commercial or financial relationships that could be construed as a potential conflict of interest.

The handling editor declared a shared affiliation with the authors at the time of review.

## Publisher’s Note

All claims expressed in this article are solely those of the authors and do not necessarily represent those of their affiliated organizations, or those of the publisher, the editors and the reviewers. Any product that may be evaluated in this article, or claim that may be made by its manufacturer, is not guaranteed or endorsed by the publisher.
